# Humidity-Responsive Photonic Films and Coatings Based on Tuned Cellulose Nanocrystals/Glycerol/Polyethylene Glycol

**DOI:** 10.3390/polym13213695

**Published:** 2021-10-27

**Authors:** Amin Babaei-Ghazvini, Bishnu Acharya

**Affiliations:** Department of Chemical and Biological Engineering, 57 Campus Drive, University of Saskatchewan, Saskatoon, SK S7N 5A9, Canada; amin.babaei@usask.ca

**Keywords:** cellulose nanocrystals, humidity sensors, chiral nematic, photonic films

## Abstract

It has been extensively reported that cellulose nanocrystals (CNCs) can represent structural colors due to their unique chiral-nematic self-assembly. However, the application of this remarkable structure does need further investigation. It has been challenging to keep the selective reflection band (SRB) resulting from the CNC structure in the visible spectrum. Herein, composition of CNC colloidal suspensions with polyethylene glycol (PEG) and glycerol (Gly) have been studied to develop humidity-responsive sensors in the form of coatings and films. The fabricated samples were characterized for their mechanical properties, optical properties, water uptake capacity, water contact angle, and surface roughness. Additionally, the chemical structure of the samples was studied with FTIR spectroscopy. The produced humidity indicators on microbial glass slides were maintained and tested in a different relative humidity range from 20% to 98% with a different color response from blue to red, respectively. The color change of the humidity sensors was reversible for several cycles. It should be noted that the color change can be detected easily by the naked eye. The water uptake test showed that pure CNC and CNC/Gly had the lowest (34%) and highest (83%) water absorption levels. The mechanical tests for CNC/PEG composites showed the highest tensile strength (40.22 MPa). Moreover, microstructural characterizations confirmed the CNC pitch formation in all the samples. Addition of the fillers increased the CNC pitch, resulting in a mesoporous film formation. These produced humidity sensors are promising candidates in food and drug packaging due to their biodegradability, biocompatibility, and cost-effectiveness.

## 1. Introduction

The importance of color in natural creatures is undeniable. Structural colors and pigments are two common forms of color in nature. There are numerous examples in nature, from animals to plants, producing and representing unique colors in the form of structural colors [1,2]. Compared to synthetic or natural dyes, which mostly come from pigments, structural colors have an entirely physical explanation, such as diffraction and refraction of light passing through the unique structures [3]. Structural colors have several advantages compared to pigments and dyes, such as stability and reversibility [4]. To some extent, structural colors have the potentials to replace synthetic dyes and heavy metal pigments due to their stimuli-responsive behavior, eco-friendliness, and sustainability [5]. Regarding these microstructures, the chiral-nematic phase of liquid crystals can represent birefringence optical properties, which are related to the isotropic, anisotropic, and cholesteric phase formation [6].

Cellulose nanocrystals (CNCs) are one the most promising materials in terms of sustainability due to their biocompatibility, abundance, and biodegradability. CNCs show an excellent photonic property due to the ability to form chiral nematic photonic films through a self-assembly process, making them considerable materials to fabricate photonic films with iridescent structural colors [7,8,9,10]. Cellulose is available in high amounts in nature, with a yearly estimated production of around 90 billion metric tons [6]. The cellulose polymer chain included d-glucopyranose units connected by β-1,4-glycosidic bonds and chains containing intramolecular and intermolecular hydrogen bonding, which plays an important role in the cellulose structure. Cellulose can be found in plants [11], bacteria [12], and tunicates [13], which are the only animals generating cellulose in earth. Sulfuric acid hydrolysis of cellulosic biomass is a well-known method for the extraction of CNCs. Nanocrystals obtained from this method have a negative surface charge due to the remained sulfate half-ester groups on the cellulose crystals [14]. Surface charges play an essential role in forming chiral nematic helical structures during the self-assembly of the CNC colloidal suspensions. CNCs can develop spontaneous self-assembly in nanoscale under a specific condition to produce a left-handed helical architecture, resulting in films with structural color properties [14,15]. Typically, the formation of a chiral-nematic structure during the CNC film-making process is impacted by electrostatic repulsions and van der Waals forces on the surface of the nanocrystals [16]. Changing the physicochemical features of the chiral nematic structure, such as pitch, can change the photonic properties of the CNC films due to change in the refraction index of the photonic structure; for example, ultrasonic treatment [17], addition of electrolytes [18], electromagnetic field exposure [19,20], evaporation rate [21], and co-assembly with water-soluble polymers or compounds [22,23,24,25,26,27], are some of the most studied methods in this field. The structural colors reflected by the chiral nematic CNCs resulted from self-assembly is entirely “green”. They can show a response to external functions, such as humidity, pressure, temperature, and electromagnetic fields in the form of visible color change [6,8,9,10]. CNC can be considered a 1D photonic crystal, and the structural color produced by the photonic crystals can be impacted by changing the particle’s size, spacing among the particles, or the size of the helicoidal pitch in response to stimulus [28,29].

**Figure 1:**
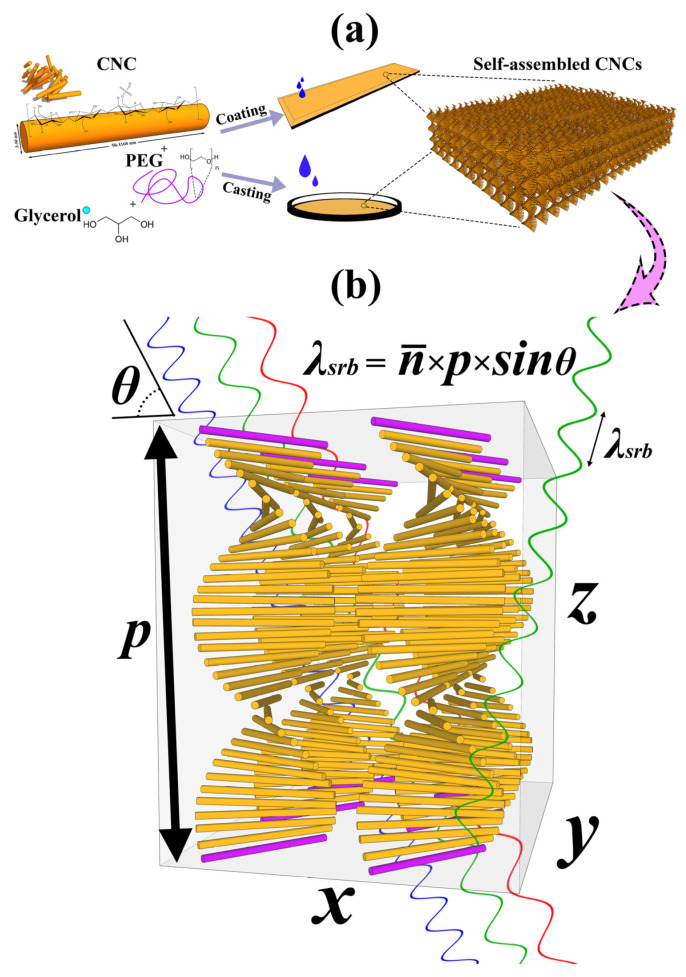
(**a**) Schematic of sample fabrication method and the helicoidal structure formation during the evaporation induced self-assembly (EISA). (**b**) Theoretical chiral-nematic arrangement for CNC photonic films and the selective reflection band illustration with modified Bragg’s law ($\lambda_{srb}$= $\bar{n}\times p\times \sin\;\theta$). The pitch is specified by *p*, the distance between the purple rods.

Various natural biodegradable and bio-based polymers from proteins, lipids, carbohydrates, polysaccharides, and their by-products have developed intelligent and smart edible films and coatings. Mainly, carbohydrate-based edible films and coatings are considered the most capable ones for food packaging and preservation [30,31,32,33]. The addition of some functional properties to these coatings and films, including sensing, antimicrobial, and UV-blocking properties, has been of interest to scholars in recent years [34,35,36]. Hence, the use of CNCs for fabricating intelligent materials has been considered in recent years [14,27]. Among these properties, responsive sensors based on CNC thin-film composites have been studied by several researchers recently [7,8,37]. However, CNC films are very brittle and water-sensitive, which makes the investigation for applied purposes difficult. Additionally, the films obtained through the evaporation-induced self-assembly (EISA) could have different structural and optical properties, because there are always significant factors in self-assembly such as surface charge energy, CNC morphology, external stimuli, and nematic energy [38,39,40]. Hence, this competition results in different pitch and, finally, different reflection bands and colors from the films. The CNC chiral-nematic structure leads to a selective reflection band (SRB) of light in Bragg’s regime when the pitch is equivalent to the wavelength of the incident light. Hence, where the Bragg condition is met, the wavelengths of the selective reflection band $\lambda_{srb}$ are given by $\bar{n}\times p\times \sin\;\theta ,$ where $\bar{n}$ is the average of ordinary and extraordinary refractive indices *(*$\bar{n}=\left(no+ne\right)/2$*), p* is the helical pitch (nm), and θ is the incident angel of the incoming light with the film surface (Figure 1b). The CNC photonic film reflects light with a similar handedness to the chiral nematic phase (left-handed), transmitting light with the opposite handedness (right-handed) [41].

Glycerol (Gly) is a simple polyol used as a plasticizer in biodegradable film-making studies, photonic coatings, and as filler to tune the pitch in stimuli-responsive CNC-based CNC materials with different glycerol concentrations [22,25,42]. Polyethylene glycol (PEG) is a polyether compound that has been widely used as a covalent modifier of biological macromolecules [43]. It should be noted that using plasticizers with low molecular weight may have some drawbacks, such as decreasing the mechanical and humidity-related properties [42]. To solve this problem in nanocomposite studies, researchers tried to use different plasticizers or a combination of several plasticizers [44]. In this regard, the mixture of glycerol and polyethylene glycol (PEG) on water vapor permeability (WVP) and mechanical properties of a nanocomposite have been studied by Park et al. [45]. According to this study, an equal combination of the Gly/PEG showed the best mechanical and WVP properties.

It is still revolutionary to achieve a homogeneous CNC photonic film with a constant, repeatable method. Additionally, obtaining the same results from stimuli-responsive applications is challenging due to the absence of an effective procedure in producing these photonic materials. Regarding finding a better application for using CNCs as an intelligence material, we report a facile method to provide stimuli-responsive chiral nematic CNC films and coatings with fast response, wide range, and reversible structural colors in response to surrounding humidity, while showing good mechanical properties, which could open a new approach for developing biodegradable materials for functional packaging. The fabricated samples were tested for their photonic responses in relative humidity, mechanical behavior, water absorption capacity, and water contact angle.

## 2. Materials and Methods

### 2.1. Material

**Figure 2:**
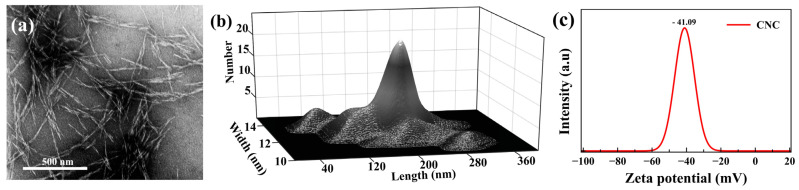
(**a**) TEM micrograph of CNC. (**b**) Width and length distribution of the employed CNCs based on TEM images. (**c**) CNC colloidal suspension surface charge.

Wood CNC in the form of gel (10 wt.%) was prepared by the sulfuric acid hydrolysis of hardwood pulp and was provided by the United States Forestry Service, Forest Product Laboratory, Madison, Wisconsin, United States. A 10 wt.% CNC gel was diluted to 3 wt.% suspension to keep the CNC suspension under the critical concentration (≈4 wt.%) [25,46] before use for making the photonic films. The morphological and size distribution information regarding the used CNC has been provided in Figure 2. Glycerol (Product number: G5516), polyethylene glycol (PEG 10 kDa, Product number: 81280), potassium acetate (CH_3_CO_2_K), magnesium chloride (MgCl_2_), magnesium nitrate (Mg(NO_3_)_2_), sodium chloride (NaCl), and potassium sulfate (K_2_SO_4_) were all purchased from Sigma-Aldrich (Canada, Ontario). All materials were used without any additional purification.

### 2.2. Preparation of CNC/Gly/PEG Samples

Since this study is an extension of a recent published work, the optimization of the fillers level was done [35]. Hence, a total of 1 g of filler (Gly or PEG) was added to 300 g of CNC suspension (3.0 wt%). The prepared suspensions were stirred for 48 h at 400 rpm at room temperature and ultrasonicated for 1 h before casting the samples. Then, 5 g of the final suspensions were cast on microbial glass slides (75 mm × 25 mm) by a syringe. Additionally, 20 g of the prepared suspensions were cast into a flat plastic Petri dish with a diameter of 10 cm and dried for 72 h at 20 °C and RH of ≈20% in an adjustable environmental chamber. By changing the amount of added Gly and/or PEG, composite films with different weight ratio of CNC/Gly/PEG were prepared. The component ratio of the studied samples is represented in Table 1.

### 2.3. Optical Characteristic, UV-Vis Spectroscopy, and FTIR Spectroscopy

A specimen’s color was determined using a colorimeter (WR10QC-8 portable colorimeter) with a CIE standard illuminant D65 and CIE chromaticity diagram pointer. Samples were placed on a black standard plate (L* = 1, a* = 0, b* = 0), and the lightness (L) and chromaticity parameters, a (red-green) and b (yellow-blue), were measured. All colors can be described by L values ranging from 0 (black) to 100 (white): negative values of a (greenness) to positive values (redness) and negative values of b (blueness) to positive values (yellowness). All measurements were performed in five replicates. POM was performed on a microscope (Olympus BH2 BHM, Tokyo, Japan) equipped with crossed polarizers to observe the samples’ birefringence properties. A selective reflection band of each sample was measured using a spectrophotometer (Agilent 8453 UV-Vis-NIR, California, United States). The film samples were placed directly in the spectrophotometer test cell, and the air was used as reference. The optical absorbance of the films was measured in the wavelength range of 200–1100 nm. For each sample, five scans were recorded to give an average peak. FTIR spectrum of the film specimens were acquired at room temperature by ATR-FTIR (ALPHA, Bruker, Berlin, Germany). The film specimens were placed directly on the ATR crystal. FTIR spectrums were measured in the wavenumber range of 4000–400 cm^−1^ at a resolution of 4 cm^−1^.

### 2.4. Humidity Responsive Tests and Water Contact Angle

The water uptake was determined based on the difference in the weight of the samples before and after conditioning at different relative humidity (RH) provided by saturated salt solutions. The test specimens were prepared on glass slides with the dimensions of 75 mm × 26 mm. They were conditioned, respectively, at RH of ≈20%, prepared by a saturated solution of potassium acetate (CH_3_CO_2_K), RH of ≈33%, prepared by a saturated solution of magnesium chloride (MgCl_2_), RH of ≈55%, prepared by a saturated solution of magnesium nitrate (Mg(NO_3_)_2_), RH of ≈75%, prepared by a saturated solution of sodium chloride (NaCl), and RH of ≈98%, prepared by a saturated solution of potassium sulfate (K_2_SO_4_), to reach a constant weight. After weighing (*m*_1_ (g)), they were conditioned in a desiccator, providing higher RH at 20 °C to achieve a higher level of water uptake by the specimens. This measurement was a continuous experiment, from desiccator number one with the RH of ≈20% to desiccator number five with the RH of ≈98%. The moisture absorption (MA) of the specimens was calculated with Equation (1).

(1) $$\mathrm{MA}=\frac{m_{2}\;-\;m_{1}}{m_{1}\;}\times 100$$

The Sessile droplet method was used for the contact angle measurements. A 5 µL droplet of distilled water was located on the film surface, and the image of the drop was captured using a drop shape analyzer (DSA30 Krüss, Hamburg, Germany). The contact angle is defined as the angle between the baseline of the drop and the tangent line at the point of contact of the water droplet with the surface.

### 2.5. Mechanical Properties

The ultimate tensile strength (UTS) is defined as the maximum tensile stress that a polymer can tolerate. Elongation at break (EB) is described as the maximum change in length of the test sample at the breaking point, and the Young’s modulus values are calculated as the slope of the elastic area of the stress–strain curve [47]. Ultimate tensile strength (UTS), and elongation at break (EB) were evaluated by the mechanical testing device (Omegadyne LC204-200, OH, United States) according to ASTM standard method D882-02. Films were cut in rectangular strips of 100 mm length and 10 mm wide. Five replicates were run for each film specimen. TS and EB were calculated by Equations (2) and (3), respectively.

(2) $$\mathrm{TS}=\frac{F_{Max}}{A_{Min}},$$

(3) $$\mathrm{EB}\;=\frac{L_{Max}}{L_{0}},$$

where *F_Max_* is maximum load, *A_Min_* is minimum cross-section area, *L_Max_* is extension at the moment of rupture, and *L*_0_ is the initial length of the specimen. Young’s modulus (YM) was calculated by drawing a tangent up to a strain of 1% of the Stress–strain curves, as shown in Equation (4).

(4) $$\mathrm{YM}=\frac{\mathrm{Stress}\;\left(\sigma \right)}{\mathrm{Strain}\;\left(\varepsilon \right)}$$

### 2.6. Microstructure, Morphology, and Surface Texture

The microstructure of the cross-sections of the produced films was studied by scanning electron microscopy (SEM). SEM was performed on a Hitachi SU8010 instrument (Tokyo, Japan), with the accelerating electron beam at a voltage of 5 kV. The sample cross-section was prepared by breaking the film specimens in liquid nitrogen and mounting them on the sample holder using double-sided carbon tape. Before imaging, the specimens were coated with a 60:40 gold:platinum alloy using a sputter coater. To evaluate the morphology of the CNC, transmission electron microscopy (TEM) micrographs were obtained on a Hitachi HT7700 instrument (Tokyo, Japan). Dilute (0.001 wt%) colloidal suspension was dispersed uniformly in water by ultrasonication, then cast onto etched copper-coated grids, stained with uranyl acetate, and air-dried before imaging. The average length and width were calculated from 50 measurements from micrographs using Image J software. The surface charge of the sample was obtained by a Brookhaven instruments (New York, NY, USA) Zeta-potential analyzer (DLS). Figure 2 shows the morphological information of the used CNC. The surface texture of the samples was characterized based on surface SEM images, reconstructed in MATLAB software with some changes according to the recently published work [48] as a 3D profile of the surface.

## 3. Results and Discussion

### 3.1. Optical and Structural Characteristics

**Figure 3:**
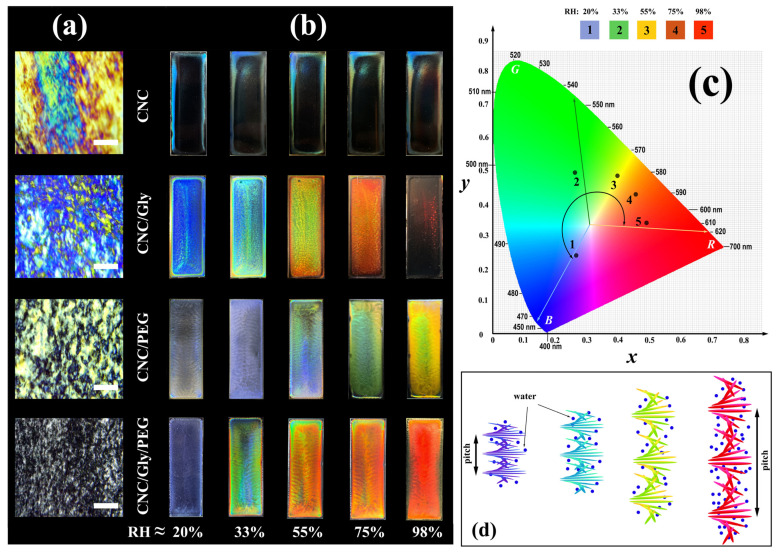
(**a**) Microscopic photographs of the produced samples in the lab environment between cross polarizer (scale bar: 200 μm). (**b**) Digital photographs of CNC, CNC/Gly, CNC/PEG, and CNC/Gly/PEG nanocomposite membranes with various structure iridescences against black background with vertical illumination of white light with changing RH *≈* (between 20 to 98). (**c**) CIE chromaticity diagram of the CNC/Gly/PEG samples based on the average colors of the sensors in different humidity. (**d**) The schematic of change in the pitch of the CNC chiral nematic structure with increasing the relative humidity pressure.

The structures of CNC composites were studied between the cross-polarizer setup with a digital microscope. Strong birefringence and colorful regions (Michel-Lévy interference color chart) [49] with random sizes and alignments were observed in the neat CNC film (Figure 3a). These domains show alignment of the CNC rods in the film that did not involve in the helix formation, which could be the result of a competition between different factors such as nematic energy, surface tensions, different evaporation rates, etc. It is observable that, by adding the fillers to the CNC films, the number of domains with Michel-Lévy interference color patterns decreased. Accordingly, for the CNC/Gly, CNC/PEG, and CNC/Gly/PEG, the obtained photos show more negligible birefringence, respectively. It seems that, by combining Gly and PEG in the CNC structure, the formation of the helicoidal blocks would be facilitated, resulting in smaller dark (isotropic) and bright (anisotropic) domains. It should be noted that these bright domains are rather randomly oriented (chiral nematic) areas that help to have more structural colorations in the matrix.

Digital photographs of the produced samples taken in the perpendicular direction $\left(\sin\;\theta =1\right)$ on a matte black background are shown in Figure 3b. For the neat CNC samples, the “coffee stain” effect [50] is recognizable to the naked eye. Water evaporates from the CNC suspension on the surface (mostly on the edge), and evaporation-driven mass transfer accumulates the CNCs to the edges of the glass slide. Finally, different thicknesses and concentrations of drying suspension give a nonuniform and imbalanced surface of film formation. The responsibility of Gly and PEG in the structure is locating between the chiral nematic layers, increasing the pitch, and providing a porous entanglement for water vapor to be absorbed. Additionally, Gly and PEG, by forming hydrogen bonds, can lubricate the change in the pitch size and increase the composite water capacity. In this regard, the produced samples were tested in different humidity levels to achieve a constant weight. According to Figure 3b, increasing the ambient from RH ≈ 20% to 98% color of the CNC composites was changed from blue to red. In particular, for CNC/Gly, the colors have been changed from dark blue, light blue, green, orange, and transparent. This procedure for the CNC/PEG resulted in a yellow color in RH ≈ 98%, which can be related to an increased interaction between PEG molecules and chiral nematic CNC structure. The obtained results for CNC/Gly and CNC/PEG humidity-responsive behavior were in agreement with similar studies [8,22]. Between the produced samples, the best humidity-responsive coverage was related to the CNC/Gly/PEG. In the CNC-based photonic film fabrication, high PEG concentration could cause the many-body interaction (here, when a macromolecule interacts with two or more CNCs) between the PEG molecules and CNCs. The many-body interaction could affect the self-assembly process due to the possible network formation between the PEG and CNC [51]. Alternatively, due to glycerol’s much lower molecular weight compared to PEG, they act as plasticizers in the composites [42], and the many-body interaction could be less likely [35]. The relatively higher water absorption in CNC/Gly and relatively lower water absorption in CNC/PEG could be solved by an equal combination of Gly and PEG, proved by the humidity responsive test. Figure 3c shows the CIE chromaticity diagram of the CNC/Gly/PEG samples based on the average colors of the sensors in different humidity. Figure 3d illustrates the mechanism of pitch change and provides an overview of how different RH affects the CNCs chiral nematic layers. The change in the pitch of the chiral-nematic layers results in different reflections in Bragg’s regime [6].

**Figure 4:**
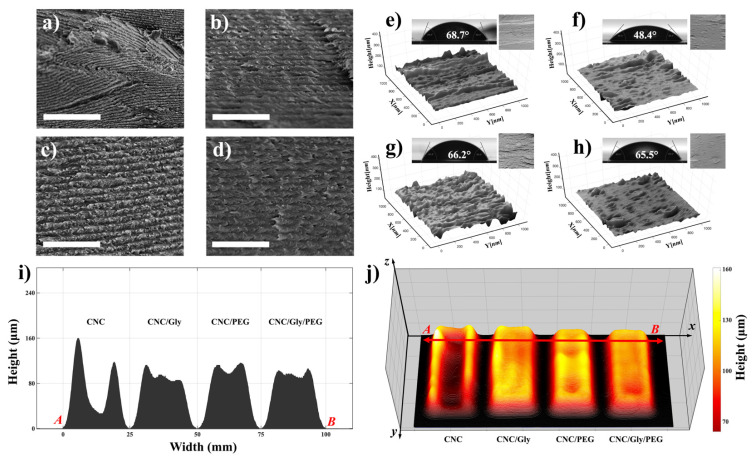
(**a**–**d**) Cross-sectional SEM micrographs of CNC, CNC/Gly, CNC/PEG, and CNC/Gly/PEG samples, respectively. The scale-bar and for the SEM images is 1 μm. (**e**–**h**) The corresponding 3D reconstructed images of the nanocomposite’s surfaces containing the water contact angle and the original SEM image as the inset for the pure CNC, CNC/Gly, CNC/PEG, and CNC/Gly/PEG, respectively. (**i**) cross-section profilometry from point A to B. (**j**) Surface topography and 3D mass distribution height map of the CNC, CNC/Gly, CNC/PEG, and CNC/Gly/PEG samples.

Figure 4a–d demonstrate SEM micrographs from the cross-section of CNC, CNC/Gly, CNC/PEG, and CNC/Gly/PEG samples, respectively. As shown in Figure 4a, for neat CNC, the formation of chiral nematic structure happened. Still, due to the competition between the enrolled factors in the development of this structure, including nematic energy and evaporation-induced self-assembly, the nematic layers were distorted. With the addition of 10 wt.% Gly to the structure, the chiral nematic structure was more ordered (Figure 4b). Additionally, PEG as a filler showed the same improvement in the chiral nematic structure. However, due to the difference in the molecular weight of PEG and Gly, the cross-section structure was slightly rough in CNC/PEG compared to CNC/Gly. Finally, the combination of PEG and Gly did not show roughness in the cross-section, and the pitch formation was seemingly smooth.

To some extent, surface geometry and morphology could change the nanocomposite’s physical properties, including water absorption. Figure 4e–h represents the 3D reconstructed surface topology in a micron dimension. As we can see in Figure 4e, pure CNC surface structure showed higher roughness than CNC/Gly. This confirms the results from the SEM cross-section of the pure CNC and the other nanocomposites; by the addition of the fillers, the CNC nanocomposites showed smooth structure. However, the CNC/PEG surface had slightly higher roughness compared to the CNC/Gly and CNC/Gly/PEG, which could be associated with the higher molecular weight of PEG than of Gly [35].

The contact angle of a water droplet is one of the promising methods to measure the wettability of the surface of materials. The insets of Figure 4e–h represent the captured water contact angle pictures and the degree of the droplet angle on the surface section of the produced pure CNC, CNC/Gly, CNC/PEG, and CNC/Gly/PEG, respectively. As shown, the pure CNC films had the highest water contact angle (68.7°), which could be associated with increased CNC–CNC interaction. CNC/Gly showed the lowest water contact angle (48.4°), related to the addition of Gly into the structure. Due to glycerol’s much lower molecular weight, it seems the addition of that made the nanocomposite more hydrophilic, which is in agreement with similar works in the literature [52,53,54,55]. CNC/PEG and CNC/Gly/PEG showed water contact angle degrees close to those of the pure CNC films.

Due to the importance of the physical properties of the produced coatings, the surface topography and profilometry of the samples were studied by 3D volumetric image processing in MATLAB in a further study. The thickness and the structure of the humidity-responsive CNC-based materials can severely impact the final color. The results are demonstrated in Figure 4i–j. According to Figure 4i, different thicknesses of the films from selected point “*A*” to point “*B*” decrease by adding the Gly and PEG to the CNC matrix. A comparison between the composites in Figure 4j shows that combining the Gly and PEG results in a more uniform and even film formation. In other words, the unity of the CNC mass distribution across the composite matrix by using the Gly and PEG simultaneously has been distributed approximately equal between the center and edges.

### 3.2. Mechanical Properties, Water Absorption, and FTIR Spectroscopy

**Figure 5:**
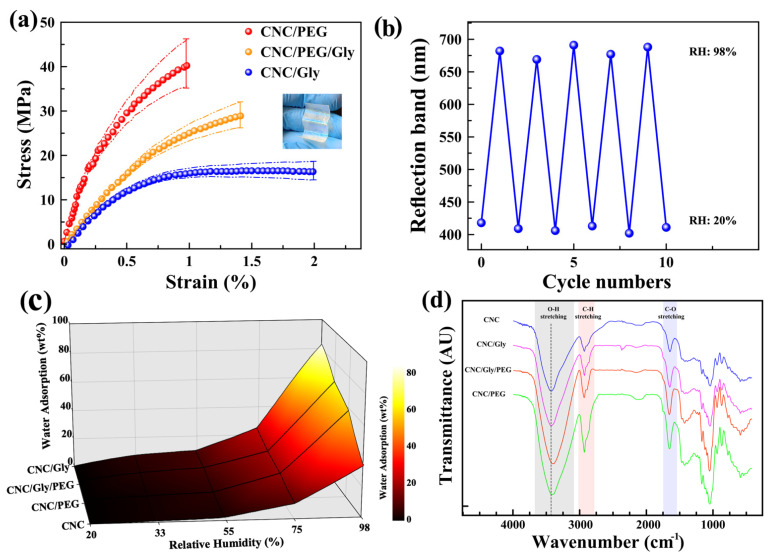
(**a**) Stress–strain curves of the CNC/Gly, (**b**) reversible conversion of the reflection band of the CNC/Gly/PEG coating by exposing alternately between RH 20% and 98% for ten cycles. (**c**) CNC/PEG and (**c**) CNC/Gly/PEG composites. (**d**) Surface plot of water uptake of the CNC, CNC/Gly, CNC/PEG, and CNC/Gly/PEG composite samples in different humidity levels. (**d**) FTIR transmittance spectra of CNC, CNC/Gly, CNC/Gly/PEG, and CNC/PEG from top to bottom, respectively.

The average stress–strain curves of the CNC/Gly, CNC/PEG, and CNC/Gly/PEG composites are shown in Figure 5a. Measuring the mechanical properties of the pure CNC films was impossible due to their brittleness. It was impossible to cut them in standard dimensions for the tensile tests to compare with the composites. Typically, CNC films have high toughness and stiffness due to their high crystallinity. Hence, adding some plasticizer fillers such as PEG and Gly could result in films with increased plastic properties due to the energy dissipation throughout the sample during the external force application [56]. According to Figure 5a, the average stress–strain curve of CNC/PEG composites showed 40.22 MPa TS (the highest) and 0.98 mm EB (the lowest). The TS for CNC/Gly/PEG was 28.89 MPa, and for CNC/Gly, was 16.33 MPa. Additionally, EB for the CNC/Gly/PEG and CNC/Gly was 1.41 mm and 1.99 mm, respectively. As shown in the inset photograph (Figure 5a), the produced CNC/Gly/PEG films demonstrated flexibility due to the combination of PEG and Gly. According to the stress–strain curves, the role of PEG in comparison to Gly is towards forming a polymeric network throughout the CNC chiral nematic structure, and the Gly’s performance is for a plasticizing effect between the composite’s components. This can be related to the difference in the molecular weight between PEG and Gly. In the study of CNC-based iridescent films, high PEG concentration could interact with more CNCs, known as many-body interaction, possibly causing a consequent network formation between the PEG and CNC [51]. Instead, due to glycerol’s much lower molecular weight than PEG, this small molecule can act as a plasticizer in the composites [42], and the many-body interaction could be less likely [35]. Hence, the mechanical behavior of the films could be affected by this phenomenon; as we can see, the CNC/PEG showed higher TS compared to CNC/Gly. As the leading composites of the study, the tensile strength of CNC/Gly/PEG has been located between the upper band (CNC/PEG) and lower band (CNC/Gly), as was expected. In terms of the role of fillers in the CNC photonic films, the obtained results from stress–strain curves were in agreement with similar past studies [22,35,57].

Any change in the structural color of photonic CNC films in different RH could be related to a change in the pitch by absorption and desorption of water vapor into the chiral nematic structure. Therefore, measuring the reversibility and hydrophilicity of the films could represent the relationship between the repeatability, water uptake, and structural color change. Figure 5b shows the cycle numbers (10 cycles) when the coating was alternatively exposed to 20% and 98% relative humidity. According to the peaks, there is a slight fluctuation in the reflection band, which could be negligible. Hence, these results confirm the excellent repeatability and reversibility of the produced sensors, and the results are in agreement with similar past studies [24,25]. Figure 5c shows the amount of water uptake by CNC, CNC/Gly, CNC/PEG, and CNC/Gly/PEG nanocomposites in different humidity environments in a colored mesh plot. The water uptake by pure CNC films was much lower than that of the other specimens, associated with less free volume and condensed crystalline structure of the neat CNC films. As we can see, with the addition of the PEG and Gly to the composite, the water absorption increased dramatically, especially in high RH levels. For example, in 98% relative humidity, the adsorbed water by neat CNC is 34%, 49% lower than the CNC/Gly. A comparison between Gly and PEG shows that Gly made the CNC structure more hydrophilic than PEG in the same weight percentage.

The transmittance FTIR spectra of the CNC films in the range of 400–4000 cm^−1^ are shown in Figure 5d. As it is shown and highlighted on the figure with grey color, the broad spectral region from 3000 cm^−1^ to 3600 cm^−1^ contains the fundamental stretching modes of hydroxyl groups (O-H). The spectrum from 3000 to 2800 cm^−1^ was attributed to the symmetric and anti-symmetric stretching modes of CH in methyl (CH3) and methylene (CH2) functional group, and 1720–1740 cm^−1^ was assigned to C-O stretching [58]. The O-H stretching band for pure CNC sample centered around 3400 cm^−1^, with the addition of fillers (Gly or PEG) slightly shifted to lower wavenumbers for three other films, indicating the increased hydrogen bond interactions in the composite film. CNC/Gly/PEG showed the highest shift to the lower wavenumbers, probably due to better interaction between the filler and CNCs. The results from this study regarding the peak shifts are in agreement with similar past studies [35,59].

## 4. Conclusions

This research studied the combination of glycerol and PEG as a filler in developing chiral nematic CNC composite for humidity sensing purposes. The results from SEM micrographs demonstrate that the composite of Gly and PEG would make an ideal combination to control the CNC pitch in films and coatings. This combination resulted in a uniform helicoidal structure and a wide range of humidity responsive in a specific time compared to the CNC/Gly or CNC/PEG composites. The surface roughness results for pure CNC show higher roughness than CNC/Gly, which was in agreement with the SEM cross-section results in the formation of a smooth structure. Regarding the water contact angle tests, the pure CNC films had the highest water contact angle (68.7°), which could be associated with increased CNC–CNC interaction. CNC/Gly showed the lowest water contact angle (48.4°) related to the addition of Gly into the structure. Based on FTIR results, CNC/Gly/PEG showed the highest shift to the lower wavenumbers on the O-H stretching band (around 3400 cm^−1^), probably due to better interaction and increased hydrogen bonding between the PEG, Gly, and CNCs. The tensile strength of the CNC/Gly/PEG was 28.98 MPa, which was higher than that of CNC/Gly and lower than that of CNC/PEG. CNC/Gly/PEG was very flexible, and the color changes in different RH were reversible. The tuned chiral nematic CNC materials are highly capable of being used in food and pharmaceutical packaging due to their sustainability and biocompatibility.

## Figures and Tables

**Table 1:** The abbreviations and the percentage of the components in the samples.

Abbreviation	CNC (wt.%)	Glycerol (wt.%)	PEG (wt.%)
CNC	100	0	0
CNC/Gly	90	10	0
CNC/PEG	90	0	10
CNC/Gly/PEG	90	5	5
